# Neuroretinitis, a great mimicker

**DOI:** 10.4103/0972-2327.41879

**Published:** 2008

**Authors:** Sunil K. Narayan, Subashini Kaliaperumal, Renuka Srinivasan

**Affiliations:** Department of Neurology, Jawaharlal Institute of Postgraduate Medical Education and Research, Dhanvanthari Nagar, Pondicherry-6, India; 1 Department of Ophthalmology, Jawaharlal Institute of Postgraduate Medical Education and Research, Dhanvanthari Nagar, Pondicherry-6, India

**Keywords:** Neuroretinitis, optic neuropathy, stellate maculopathy

## Abstract

Neuroretinitis is a less-known clinical entity, which can be funduscopically confused with papillitis or papilledema and with hypertensive, renal and infiltrative retinopathies as well as with retinal vein occlusion or anterior ischemic optic neuropathy.

**Report:**

Two young patients presented with sudden onset of blurring of vision. Ophthalmic evaluation revealed a characteristic picture of neuroretinitis. Detailed study of the cases failed to indicate any specific etiology, thereby suggesting the diagnosis of idiopathic neuroretinitis. Although funduscopy showed marked inflammatory changes in the retina and optic nerve head, the recovery following medical treatment was remarkable.

**Comment:**

Familiarity with the fundus picture and awareness of the specific causes of neuroretinitis among neurologists and physicians may enable a prompt clinical diagnosis, avoidance of expensive brain imaging studies and early referral for appropriate and effective therapy. A brief review of the literature is presented suggesting a need for further studies to establish specific environmentally determined etiological factors such as infections and the effectiveness of the current modalities of treatment.

## Introduction

Neuroretinitis is a type of optic neuropathy characterized by an acute unilateral visual loss in the setting of optic disc swelling accompanied by hard exudates characteristically arranged in a star shape around the fovea.[1] In fact, it is classified as one form of optic neuritis, the other forms being the more common retrobulbar neuritis and papillitis. Funduscopically, neuroretinitis is often confused with hypertensive, renal and infiltrative retinopathies as well as with papillitis, papilledema, anterior ischemic optic neuropathy and retinal vein occlusion. It is an entity that physicians, pediatricians and neurologists are poorly exposed to, since the diagnosis and management are almost exclusively performed by the ophthalmologist. Pathogenesis and etiology of neuroretinitis are distinctly different from other funduscopically resembling conditions encountered by neurologists very often during their training and clinical practice; further, these have obviously different principles of management and prognosis. Prognosis for visual recovery is reported to be excellent, althoughnot uniform.[2] Our objective is to present two typical cases of neuroretinitis and highlight the need for neurologists to distinguish it from optic neuritis or retinal disorders that it closely mimics.

## Case Reports

### Case 1

**Figure 1:**
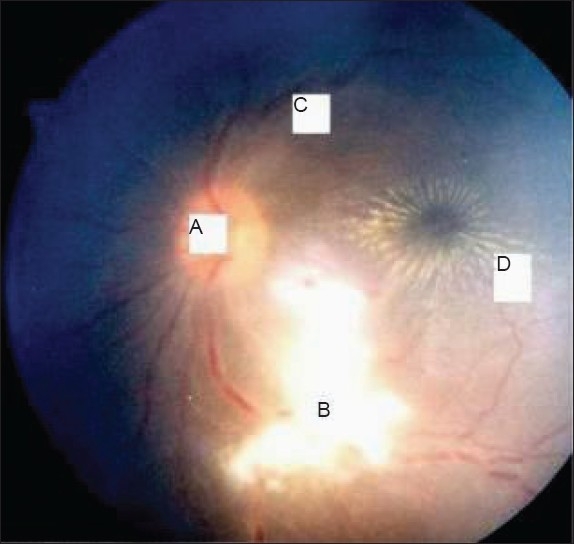
Case 1- Neuroretinitis with (a) swelling of optic nerve head,(b) subretinal exudates, (c) dilated and tortuous veins and (d) classicalstellate maculopathy

An 18-year-old female presented with painless blurring of vision in the right eye for 20 days preceded by a mild fever. There were no other constitutional symptoms; history of tuberculosis; or contact with dogs, cats or other pet animals. On examination, a grade II relative afferent pupillary defect with a best-corrected visual acuity of 6/24 in the right eye and 6/6 in the left eye was noted. A slit-lamp examination disclosed a cellular vitreous humor but clear anterior segment on the right. Fundus examination of the right eye revealed an optic disc with blurred margins and hyperemia and star-shaped hard exudates distributed in the macula. There was also deep subretinal exudation along the superotemporal vessels suggestive of vasculitis [Figure 1]. Color vision was impaired in the right eye than that in the left eye. Visual field testing revealed a centrocaecal scotoma in the right eye. Visual evoked potential (VEP) showed a latency of 105 ms on the affected side and a relative decrease in the amplitude of the waveform compared to the left. Electroretinogram (ERG) was normal. Fluorescein angiography revealed a leakage in the disc and along the superotemporal vessel; no aneurysmal dilatations of arterioles and no leakage at the macula were noted. Ultrasonography of the right eye showed widening of the optic nerve sheath with a mild elevation of 1 mm of the optic nerve head. The other eye was unremarkable. The remaining cranial nerves and the results of neurological examination were normal. There were no meningeal signs. There was no lymphadenopathy, rashes and the result of the remaining physical examination was also normal. ELISA for cysticercosis, tuberculosis, Lyme disease, leptospirosis, brucellosis, toxoplasmosis and toxocariasis and Weil-Felix test showed negative result. Serum HIV, HBsAg, VDRL, Herpes, CMV and Rubella serology, Paul Bunnel test and cold agglutinins showed negative result. The serology for Bartonella henselae, which is responsible for cat-scratch disease (CSD), could not be performed. Antinuclearand antiphospholipid antibodies revealed negative result. The findings of computed tomography, MRI and MR venogram of the brain were normal. Following a diagnosis of idiopathic neuroretinitis, the patient was administered oral prednisolone for three weeks. The vision improved to 6/12 after a period of two weeks. Six months later, the deep exudates remained but otherwise the fundus became normal with the return of visual acuity to 6/9. No neurological symptoms supervened at any time. The patient remained asymptomatic during a follow-up period of the next two years.

### Case 2

**Figure 2:**
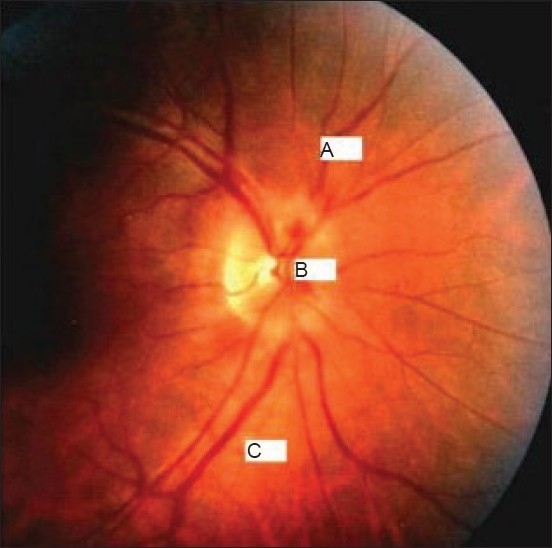
Case 2- Neuroretinitis with edematous but pale disc and blurred margins; (a) dilate d and tortuous veins, (b) papillary hemorrhageand (c) focal and perivenous edema

A 26-year-old female presented with a progressive decrease in vision in the right eye for three weeks. There was no history of trauma or contact with cats or other pets. There was no fever or other symptoms at the onset of this problem. On examination, the best-corrected visual acuity was 6/12 in the right eye and 6/6 in the left eye. The optic disc was hyperemic with blurred margins. The veins were dilated and tortuous. At the initial presentation, a slit-lamp examination with a 90-D lens confirmed the presence of a mild macular edema. The retinal periphery was normal [Figure 2]. Patient developed stellate maculopathy three weeks later. Color vision was severely impaired in the right eye. VEP showed a lower amplitude on the right side with a latency of 107 ms but ERG was normal. Fluorescein angiography showed diffuse leakage from the disc but not from the macula or retina. Central fields of this patient reveale d a centrocaecal scotoma in the right eye and normal left eye fields. A systemic examination of the patient was normal. Ultrasonography of the right eye showed an elevationof 2 mm of the optic nerve head. The other eye was unremarkable in all aspects. Radiological examination of he skull and paranasal sinuses was normal. MRI of the brain and MR venogram were within normal limits. The results of all blood tests, including serological tests for syphilis, Lyme disease, toxoplasmosis and toxocariasis and the remaining serology, similar to that in case 1 was negative. A general physical examination of the patient was also normal. Idiopathic stellate neuroretinitis was diagnosed. No specific treatment was administered in the light of relatively minimal impairment of the visual acuity. Six weeks after the onset, the optic nerve swelling had decreased but the macular star-shaped figure persisted. The vision had returned to normal, and she continued to be asymptomatic over a follow-up period of one year.

## Discussion

Neuroretinitis presents with sudden visual loss, swelling of the optic disc, peripapillary and macular exudates that may occur in a star-shaped pattern and cells in the vitreous.[1] It affects persons of all ages, more often in the third and fourth decades of life, with no gender predilection.[2,3] Visual acuity at the time of initial examination ranges from 6/6 to light perception (PL). The most common field defect is caecocentral scotoma, but central scotomas, arcuate defects and even altitudinal defects may also be present. A relative afferent pupillary defect is present in most patients, unless the condition is bilateral. VEP is useful in the setting of suspected multiple sclerosis in which there is a prolongation of latency of the P100 wave and a decrease in amplitude. The amplitude of VEP may be abnormal in neuroretinitis too. ERG is usually normal since it assesses the functional integrity of the retinal layers and hence grossly normal in a disorder involving ganglion cells and optic nerve, such as neuroretinitis. An early defect in color vision is common as one would expect in a disease affecting ganglion cells and the macula.

The degree of the optic disc swelling ranges from mild to severe, which depends, in part, on the timing of the first examination. In severe cases, splinter hemorrhages may be present. A macular star-shaped figure composed of lipid (hard exudates) may not be present when the patient is examined immediately after the onset of visual symptoms, but tends to become more prominent as the optic disc swelling resolves.[4] The posterior inflammatory signs consisting of vitreous cells and venous sheathing as well as mild anterior uveitis may occur as noted in one of the cases encountered by us.

Diagnostically, the fundus picture may be confused with a few other entities. Papillitis, papilledema, central retinal vein occlusion and anterior ischemic optic neuropathy are the close mimickers funduscopically.[5] Differentiation, however, is not difficult by performing detailed ophthalmic, nervous system and general examination. Table 1 illustrates the differentiating features between these conditions.

**Table 1:** Differentiating features between neuroretinitis and other funduscopically resembling entities.

	Neuroretinitis	Papillitis	Papilledema	CRVO*	AION#
Visual acuity severe	6/60–6/12	Light perception	No visual loss to 6/12	Moderate to severe visual loss	Moderate to impairment
Pupillary reactions	Relative afferent papillary defect (RAPD+)	RAPD+	Normal	Normal/RAPD+	RAPD+
Laterality	Unilateral, rarely bilateral	Unilateral/bilateral	Always bilateral	Unilateral	Typically unilateral
Eye pain	Nil	Pain especially on up gaze	Nil	Painless	Painless
Visual fields	Centrocaecal scotoma	Central/centrocaecal scotoma	Enlarged blind spot	Normal	Altitudinal field defects
Color vision	Severely impaired, disproportionate to visual loss	Severely impaired, disproportionate to visual loss	Normal	Normal	Diminished in proportion to level of v/a
Systemic symptoms	Fever, rash	Weakness of limbs	Headache, vomiting, Cushing's effect, abducens palsy	Chemosis, puffiness of eyelids	Headache, scalp tenderness, jaw claudication, polymyalgia rheumatica
Fundus findings	Disc hyperemic with swelling of 2D, macular star figure	Disc swelling rarely above 2D, venous engorgement and hemorrhages less marked	Disc swelling frequently higher, upto 8–9 D, more venous engorgement, macularstar may develop	Disc edema, macular star along with hemorrhages and soft exudates	Pale disc, edema
Fluorescein Angiography	Leakage from disc and peripapillary retina	Leakage from disc and peripapillary retina	Leakage from disc and peripapillary retina	Shows areas of capillary nonperfusion	Unequal choroidal filling in arterial retina
VEP	Decrease in amplitude, increase in latency	Decrease in amplitude, increase in latency	Normal	Normal or with reduced amplitude	Decrease in amplitude
Specific associations	Syphilis, cat- scratch disease, Lyme disease	Multiple sclerosis	Hyper viscosity syndromes and prothrombotic states	hypertension, diabetes, glaucoma	Giant cell arteritis, hypertension, diabetes
Prognosis	Good	Good	Good with relief of increased ICT	Often depends on initial visual acuity	Poor

* Central Retinal Vein Occlusion

# Anterior Ischemic Optic Neuropathy

The etiopathology of neuroretinitis is obscure. Neuroretinitis is thought to be an infectious or immune-mediated process that may be precipitated by a number of different agents.[2] Commonly associated with an antecedent viral syndrome, in up to 50% of the cases, viruses are seldom cultured from vitreous and aqueous humor and CSF of such patients, and the serological evidence of a concomitant viral infection is usually lacking. Proposed causative viral agents include herpes simplex, hepatitis B, mumps and the herpes viruses associated with the acute retinal necrosis syndrome. Other common infections that cause neuroretinitis are CSD, spirochetosis especially syphilis, Lyme disease and leptospirosis. Presumed etiologies for neuroretinitis also include toxoplasmosis, toxocariasis and histoplasmosis.

The pathogenesis of neuroretinitis is associated with the direct involvement of the optic nerve fibres by the infectious process or the inflammation leading to edema and fluid exudation from the inflamed cellular area of the peripapillary retina. Since macular exudates result more likely from the primary optic nerve disease, rather than from the inflammation of the retina, the idiopathic variety is also called as ‘idiopathic optic disc edema with a macular star’ rather than ‘neuroretinitis’.[6] When optic disc swelling and macular star are associated with focal or multifocal inflammatory lesions in the retina (retinitis), especially if an infectious cause is documented, the term ‘neuroretinitis’ is indeed unquestionable. The macular exudates may not develop until two weeks after the onset; hence, the need to reexamine patients with acute papillitis with a normal macula within two weeks for the development of a macular star.

## Variants of neuroretinitis

In the absence of a proven etiology to the disease, the condition is diagnosed as Leber's idiopathic stellate retinopathy.[7] It is a diagnosis of exclusion made after ruling out other known causes of neuroretinitis. It occurs most often in healthy young subjects presenting with acute unilateral visual loss. Although treatments with systemic steroids have been attempted, there is no definite evidence that such treatment alters either the speed of recovery or the ultimate outcome.[8] The prognosis is usually good, with a spontaneous resolution within 6–12 weeks, although the macular star-shaped structure may persist beyond this period. Diffuse unilateral subacute neuroretinitis (DUSN) is a related condition thought to be caused by one or more types of helminths,[9] probably by a motile worm. In approximately 25% of the cases, a worm is visualized during the eye examination. Laser treatment for killing the worm is the only reliable way to cease the progression of this disease.[10] The most characteristic feature of idiopathic retinal vasculitis and aneurysms associated with neuroretinitis (IRVAN) syndrome[11] is the presence of macroaneurysms in the retinal and optic nerve head arterioles. Typically noted in young, healthy, female subjects, this bilateral condition is not associated with any systemic abnormalities and its etiology remains unknown. Another association is with the CSD[12] observed in young, otherwise healthy individuals with fever, malaise, lymphadenopathy and a history of exposure to or puncture wounds by cats, with neuroretinitis as a characteristic clinical feature, caused by the organism *Bartonella henselae*. Some evidence even indicate that Leber's neuroretinitis is nothing but a manifestation of CSD, but the extent of association remains to be determined. AIDS-associated CSD neuroretinitis may additionally have conjunctival and retinal bacillary angiomatosis. Although a self-limiting disorder, systemic corticosteroids with or without systemic antibiotics have been reported to be effective in this condition.[13] Azithromycin, ciprofloxacin, rifampicin, parenteral gentamicin, or trimethoprim-sulfamethoxazole have been found to be effective in immunocompromised patients. A bilateral neuroretinitis with severe anterior uveitis can occur in syphilis and other spirochaetal infections, such as borreliosis and leptospirosis, where meningeal and cerebral involvement may coexist. There is now a consensus that multiple sclerosis is one condition that is ***not*** associated with neuroretinitis,[2] although anterior and retrobulbar neuritis are intimately linked to multiple sclerosis. There is no increased risk of development of multiple sclerosis, in neuroretinitis patients. Thus, when an acute optic neuropathy is diagnosed as neuroretinitis rather than anterior optic neuritis, it substantially alters the neurological prognosis, despite some anecdotal reports of patients with 'multiple sclerosis who developed ‘neuroretinitis’.[14] The presence of a macular star-shaped structure militates strongly against subsequent development of multiple sclerosis.[5] Visual and associated neurological symptoms of neuroretinitis attest the fact that this is a disease of both the retina and contiguous neuronal elements.

In the two cases discussed above, there was no confirmed etiology despite extensive evaluation; hence, a diagnosis of Leber's idiopathic stellate neuroretinitis would be appropriate. The disease is self-limiting but patients are often treated empirically with steroids in the acute phase. In this idiopathic variety, a general prognosis for visual recovery is good, as was the case with our patients; however, there are a few reports of severe residual visual loss a well. In the secondary forms wherein there is an identified or strongly associated infectious agent, specific therapy against the organisms along with steroids appears justifiable from anecdotal reports. One form of specific secondary neuroretinitis produced by herpes virus infection can result in blindness from severe necrotizing neuroretinitis. No reliable data is available on any acute phase prognostic markers for the eventual visual outcome. Patients with recurrent attacks may not experience good recovery of optic nerve function.

## Conclusions

Neuroretinitis should be distinguished from several funduscopically confusing conditions. The time course, presence or absence of pain, pattern of visual loss (particularly visual field defects) and funduscopic appearance help to differentiate it from the common forms of optic neuropathies[15] such as papilledema, ischemic optic neuropathy, optic neuritis, compressive lesions, toxic/nutritional deficiencies, and hereditary forms; though, neuroretinitis can mimic any of these entities and pose a diagnostic challenge even to a well-experienced clinician. However, the extent of diagnostic investigations in neuroretinitis should be based on the presence or absence of associated constitutional symptoms and corroborative evidences. In the idiopathic variety, the patient is most likely to recover vision within weeks to months. Most of the current information regarding this entity has been drawn from observations from small series and anecdotal reports. Although a few well-known etiological factors have been identified, they are by themselves not common conditions (e.g., CSD) and many of the reported cases do not have uniform data. The role of steroids as a treatment remains unclear in both the primary and secondary types. Hence, a prospective multicentric registry systematically taking into account the etiological factors and outcome of the standardized treatment may provide a better insight into the etiopathology, diagnosis and management of neuroretinitis.
